# Transferability of the first-mover advantage to individual sports: a conceptual and theoretical analysis

**DOI:** 10.3389/fspor.2026.1635647

**Published:** 2026-02-18

**Authors:** Florian Riegler, Frank Daumann

**Affiliations:** Chair of Sports Economics and Health Economics, Friedrich Schiller University Jena, Jena, Germany

**Keywords:** competition, innovation, leadership position, specialization, success in sports

## Abstract

In this paper, we examine the transferability of the theory of the first-mover advantage to national sports associations in individual sports for the first time. The conceptual analysis examines the replicability through a comparative analysis, a theoretical modeling, and a case logic across three approaches. The comparative analysis detects a fundamental possibility of transferring the first-mover advantage, considering macroeconomic control variables and a specific athlete assignment process to reflect structural differences between business organizations and national individual sports associations. While the theoretical modeling demonstrates that the individual first-mover advantage components can generally be transferred to national individual sports associations, the case logic simulates how plausible first-mover effects could occur in individual sports and reveals highly relevant effects to the reality of national individual sports associations. The findings serve as an important basis for future studies to explore a potential first-mover effect in individual sports and derive success-focused implications for action.

## 1 Introduction

Over the past few decades, sport has developed into a highly globalized, professionalized, and commercialized industry. Its expansion as a global business and a political/diplomatic resource (1) is reflected, for example, in the global revenue of the sports industry, which totaled 2.65 trillion US dollars in 2023 (2). Accordingly, the sports industry is also the ninth largest industry in the world, ranking ahead of the oil and gas industry. As a result, professional athletes have become internationally recognized commercial icons, with annual earnings from salaries and sponsorship contracts reaching peaks of over 100 million US dollars (3). Due to the sharp surge in financial volume within elite sport, both the intensity of competition and the pressure on sports organizations to professionalize are increasing (4). Consequently, the relevance of success in sport is rapidly increasing as well. Analyzing success factors in sport is therefore an essential research area, with more and more parallels to the business world also being investigated due to the developments described. Thus, previous research has repeatedly applied economic theories used in business to sport to explain, predict, and better understand successful behavior in the field of sport [e.g., agency theory (5), behavioral economics (6), human capital theory (7), network effects (8), resource-based view (9–12), stakeholder theory (13, 14), statistical discrimination theory (7)]. In addition, innovations also play an important role in many sports [see (15–19) for more details].

In 2021, a new academic discussion emerged when Daumann et al. (20) were the first to examine the founding date of national sports associations as a potential success factor in international competition. This new approach transfers the theory of the “first-mover advantage”, well-known from competition and innovation economics to the field of sport and questions its role as a potential success factor. In business, the first-mover advantage describes the ability of a pioneer company to achieve long-term competitive advantages, such as a superior market position, higher market shares or higher profits, by tapping into a new market (21). Further research by Daumann et al. (22, 23) reveal empirical evidence of a strong effect of the first-mover advantage at association level as a success factor for the team sports soccer and rugby. These findings provide initial evidence of shared management characteristics between the sporting and business fields.

In this paper, we seek to further investigate the evidence for this overarching management parallel by deepening the understanding of the transferability of the first-mover advantage to the field of sports. While current research on the first-mover advantage in sport has so far been solely limited to team sports (20, 22, 23), we are analyzing the applicability of a transfer in a conceptual paper to the level of national sports associations in individual sports. This theoretical approach serves to pick up on the existing research gap and to evaluate the possibility of applying the theory to individual sports. By doing so, we want to explore if this economic theory also plays a role in individual sports to explain success. The relevance and basis of our work is based on various perspectives. For example, national individual sports associations have similar product types and production objectives as service companies, whereby numerous studies in academic literature provide evidence of a first-mover advantage in the service sector (24–26). Furthermore, there is scientific evidence that early market entry is crucial for success in international markets, particularly for business organizations with limited resources (27). National individual sports associations are in a similar situation, as they are limited to their own country's human capital stock in terms of essential resources such as their talent pool, while at the same time competing internationally. Additionally, as already explained, there is empirical evidence for a first-mover effect in team sports, which at first glance suggests that the theory can be transferred to individual sports too. However, this assumption is not entirely tenable, as there are significant differences in sport production between individual and team sports. Moreover, the conceptual transferability of the theory to team sports has not yet been systematically analyzed. Nevertheless, empirical evidence in team sports reinforces the need to fundamentally examine the theoretical transferability to individual sports. Furthermore, individual sports such as tennis and golf reveal relevant structural characteristics for an existing first-mover advantage, as their world rankings are predominantly dominated by athletes from historically established associations (28, 29).

The objective of this conceptual work is to examine if and how the theory of first-mover advantage can be applied to national individual sports associations. The aim of this study is therefore to answer the following research question: Is the theory of the first-mover advantage transferable to individual sports and, if so, which aspects need to be considered in this context?

The findings should provide insight into whether the first-mover advantage can be examined as a potential success factor in individual sports in future research. Given the initial situation outlined above, which demonstrates the relevance of this paper, it appears particularly significant given the increasingly competitive global environment in elite sports. As the global competition in elite sports intensifies, knowledge about potential success factors in individual sports can lead to a competitive advantage for national individual sports associations in international competition.

## 2 Literature review

The literature review serves as a theoretical foundation for the following analysis and classification of the first-mover advantage in individual sports. Accordingly, this section outlines the current state of research on success factors in individual sports. Since the first-mover advantage has so far only been studied in team sports, we also highlight economic differences between individual and team sports to consider the differences during the conceptual analysis.

### 2.1 Success factors in individual sports

Due to the large number of individual sports disciplines, the Olympics are the world's largest individual sports event. At the same time, the sporting success of nations has been extensively studied on the basis of major global sporting events such as the Summer or Winter Olympics [e.g., (30–37)], revealing that in GDP [per capita] (30, 32–36) and population size (30, 32, 34), two independent macro-economic variables play a decisive role in sporting success. Accordingly, more than 50% of international success in sport can be explained by these two macro-level factors (38, 39). However, studies on success factors at major multisport events such as the Olympic Games provide only limited conclusions for individual sports, as apart from a few exceptions [e.g., (31)], they also consider team sports.

Furthermore, there are several studies that deal with sports policy factors contributing to international sporting success [e.g., (38–41)]. Yet, the results are not necessarily applicable on individual sports either, as these studies do not distinguish between associations in individual and team sports.

Research that has focused specifically on success determinants in individual sports has either concentrated on specific sports [e.g., tennis (42), swimming (43) or golf (44)] or analyzed success parameters within major sporting events (31). In tennis, GDP per capita and population size are identified by De Bosscher et al. (45) as the two most important factors explaining international success. Further empirical evidence supports the importance of top-level coaching (42, 46), professional tennis infrastructure (46, 47), financial resources of associations (42, 47), or home advantage (48) in tennis performance. Following Birto (49) home advantage also plays a crucial role in Judo, while in golf, similar to tennis, success is influenced by skillfull coaches (44, 50), effective training programs (44), and association structures (44). In swimming, there is substential evidence that junior level success is a decisive factor in reaching world class level (43, 51).

According to De Bosscher et al. (38), international sporting success factors can be divided into three categories, being the macro level, meso level, and micro level. While macro level factors include the social and cultural environment in which people live, meso level factors relate to the sports strategies and policies of individual nations. The micro level contains factors that are directly related to the individual athlete and their immediate environment. However, these levels are not rigid, as they interact with each other, leading De Bosscher et al. (38) to identify an overlapping category of influencing factors between the macro and meso levels. This additional overlapping category covers the environmental aspects of the sports system such as the country's sports culture, the role of the mass media or the private sector as success factors.

Based on the categories defined by De Bosscher et al. (38), Table 1 provides an overview of researched success factors in individual sports while assigning them to the respective categories.

**Table 1:** Overview of investigated success factors in individual sports.

Success factors at macro level	Success factors at the overlapping level between macro and meso level	Success factors at meso level	Success factors at micro level
Average wage – Varmus et al. (52) Country size – De Bosscher et al. (45) GDP [per capita] – De Bosscher et al. (45) – Forrest et al. (31) – Varmus et al. (52) Human Development Index – Vyas (53) Population size – De Bosscher et al. (45) Religion – De Bosscher et al. (45)	Elite sport schools – De Bosscher & De Croock (54) Home advantage – Brito et al. (49) – Forrest et al. (31) – Koning (48) Mass media – Brouwers et al. (42) – Wijk (55) Private sport academies – Brouwers et al. (42) – Brouwers et al. (46) Sponsors – Brouwers et al. (42) – Fry et al. (56) – Wijk (55) Sports culture – Brouwers et al. (42)	Educational concept for coaches – Brouwers et al. (42) – De Bosscher & De Knop (47), cited in De Bosscher et al. (45) Financial resources of associations – Brouwers et al. (42) – De Bosscher & De Knop (47), cited in De Bosscher et al. (45) No. of prof. tournaments hosted – Crespo et al. (57) – Filipcic et al. (58) – Reid et al. (59) – Varmus et al. (52) Professional association [infra-]structure[s] – Brouwers et al. (46) – De Bosscher & De Knop (47), cited in De Bosscher et al. (45) – Mattsson et al. (44) Top-level coaching – Brouwers et al. (42) – Brouwers et al. (46) – Kitching & Campbell (50) – Mattsson et al. (44)	Aerobic power – Neumayr et al. (60) Anaerobic power and capacity – Chaabène et al. (61) Athlete's belief system – Newton & Duda (62) Athlete's lifestyle – Aman et al. (63) Athlete's motivational patterns – Schmid et al. (64) Athlete's motor abilities – Turković et al. (65) Athlete's physical fitness – Chaabène et al. (61) – Guidetti et al. (66) – Son et al. (67) – Turković et al. (65) Athlete's psychological skills – Lundkvist et al. (68) Athlete's technique – Hébert-Losier et al. (69) School culture – Brouwers et al. (42) Social capital – Novak et al. (70) Success at junior level – Li et al. (71) – Yustres et al. (51) – Yustres et al. (43)

Source: Own table.

As can be seen, the current state of research is characterized by a large number of studies that have examined success factors in individual sports across all categories.

### 2.2 Comparing sport production: economical differences between individual and team sports

Since previous research in sports only applied the theory of the first-mover advantage to team sports, it is important to outline the economic differences in sports production between individual and team sports. Accordingly, there are fundamental differences in terms of agents, competitive situation, and marginal productivity, which are highlighted in Table 2.

**Table 2:** Economic characteristics of individual and team sports.

Economic characteristics	Individual sports	Team sports
Agent	Individual athlete as the sole agent	Team as an agent
Competitive situation	Athletes compete with all other participants in the competition	Athletes cooperate within the team; teams compete against each other in competitions
Marginal productivity	The athlete's marginal productivity solely depends on his or her own effort	The athlete's marginal productivity depends on his or her own effort and that of the team members
Examples	Badminton, boxing, golf, judo, skiing, swimming, table tennis, tennis, track and field	American football, baseball, basketball, cricket, soccer, handball, hockey, ice hockey, rugby, volleyball

Source: Own table based on Daumann (72).

In theory, the sporting result in team sports is a collective good that benefits all team members equally, regardless of their individual contribution (73). At the same time, it is not possible to determine exactly the individual contribution of a team member to the overall performance (73). Factors influencing sporting performance can be divided into club-external determinants [e.g., fan behavior or media attention], club-internal determinants [e.g., monitor, playing system or training conditions], and the individual playing strength of the team members, which is based on their potential [strategic: inherent talent and experience; operational: fitness and current form] and the extent to which they fulfil their potential [in particular motivation] (74). However, as the individual contribution of each athlete to the overall result is not measurable, the resulting information asymmetry leads to two central problems in the production of sporting performance in team sports, namely a lack of willingness to cooperate and moral hazard. While a lack of willingness to cooperate reflects the tendency among athletes to exhibit free rider behavior, moral hazard reveals trough an athletes' lack of willingness to perform. These affect operational potential and the ability to reach full potential [e.g., in the form of a decline in performance and motivation], which in turn can affect sporting performance (72). An incentive mechanism that is a compensation structure that promotes cooperation (73) offers a possible solution to address these issues. Accordingly, numerous empirical studies have confirmed a significant positive correlation between compensation and sporting performance (75–77). Furthermore, control mechanisms offer an opportunity to reduce information asymmetry. Such control mechanisms can, for example, be implemented through the coach, who can take on this supervisory role by monitoring the performance of individual team members and taking it into account when deciding on the team line-up. By determining the tactical system or making in-game decisions, coaches have additional key functions in the production of sporting performance (73). In team sports, team structure also influences performance, whereby, for example, a heterogeneous level of experience or a homogeneous market value structure within the team has a positive effect on overall performance (78). However, adverse selection causes a strategic problem in team formation (72). This is because information asymmetries can arise when new athletes are signed by clubs, since certain characteristics of the athlete remain hidden regarding their willingness or ability to perform and therefore represent hidden characteristics. To reduce this information asymmetry and to sign the best athlete for the corresponding position within the team, scouting, screening [e.g., medical examination or performance diagnostics] or self-selection [e.g., by offering highly performance-related contracts] are good strategies. Furthermore, a club can step up its youth program, which would allow the club to become familiar with the characteristics of the talented players through its training. Alternatively, a player could send signals about their own performance [signaling], for example, through recommendations (72).

In individual sports, unlike in team sports, the athlete acts as the sole trader while competing against all other athletes (79). Consequently, the marginal productivity of the athlete's performance depends solely on their own effort. As a result, the relative strength of athletes also plays a role in individual sports, whereby an increase in an athlete's strength in relation to that of other athletes automatically results in ranking effects (72). To maximize the demand for an individual sports competition, the tournament organizer, who in theory is often considered to be profit-maximizing, should primarily consider the quality of the participants and their effort to win (79), and seek to maximize the input and productivity of the athletes (80). This can be achieved through an incentive system that is tailored to the respective level within the participant field. Accordingly, the athletes' effort also plays a role when organizing sporting events in individual sports. In a symmetrical winner-takes-all competition, with a balanced performance level, the chances of winning depend on the effort of the athletes. A marginal increase in effort can enhance the chances of winning, which in turn increases the level of effort. However, as the number of participants grows, individual effort decreases due to the lower probability of success, while the overall level of effort increases (79). In asymmetric competitions with multiple participants, there is a significant performance gap between the athletes, which at first glance does not seem to promise great excitement. Studies show the superstar effect, whereby the participation of a clearly superior athlete reduces the effort of the remaining participants [e.g., (81–83)]. To promote the athletes' efforts through the incentive system in the best possible way, Szymanski (79) recommends a small prize gap between the individual ranking positions for events with a heterogeneous performance level of the participants, while a balanced field of participants is more strongly motivated by a large prize gap.

## 3 Theoretical analysis of the transferability of the first-mover advantage to individual sports

The following section forms the core of this research work by examining the transfer of the theory of the first-mover advantage to individual sports using a conceptual research design in form of a theory adaptation.

Theory adaptation represents a conceptual approach that aims to expand the scope of application of an existing theory or concept by introducing a new theoretical lens (84).

This adaption is applied through comparative analysis, theoretical modeling, and case logic in three specific approaches.

The comparative analysis provides a comparison of the structural differences between business organizations and national individual sports associations. This systematic review enables to analyze respective conditions and structures that could influence the transferability of the theory of first-mover advantage to national associations in individual sports. The aim is to highlight any necessary adjustments that may be required in the context of transferability as well as to identify potential exclusion criteria that could prevent the applicability. The comparative analysis therefore provides the contextual basis for the subsequent theoretical modeling and explores if the structural differences between business organizations and national individual sports associations contain any exclusion criteria for the transfer.

Within the theoretical modeling, we explore the potential transfer from a first-mover advantage-oriented perspective. This entails a systematic review of the individual components of the first-mover advantage to investigate if they can theoretically and plausibly be transferred to the new context. This second analysis therefore serves to reveal if the individual first-mover advantage components can be adapted to national individual sports associations.

Based on the case logic, we would like to verify the plausibility of the adapted theory through a conceptual generalized illustration. The aim is to work out how the components of the first-mover advantage could be effective and visible in the context of national individual sports associations. The illustration of potential effects provides important insights into the possible relevance of the theory for national individual sports associations.

In the context of a new theoretical lens, the combination of comparative, theoretical, and illustrative analysis forms a coherent structure to analyze the transferability of the first-mover advantage to the national association level in individual sports.

### 3.1 Comparative analysis: critical review of the differences between business organizations and national individual sports associations and their impact on the transferability

There are numerous studies within the academic literature that discuss the structural and functional differences between sports organizations and business organizations [e.g., (85–87)]. The most used principles for discussion are the constitutive (85, 86) and economic (86) characteristics of non-profit sports organizations, which set them apart in their organizational attributes from for-profit organizations (86). These characteristics therefore form the conceptual basis for the following comparative analysis. In this context, we only consider the consecutive [“voluntary membership”, “orientation towards member's interests”, “democratic decision-making structures”, “voluntary work”] and economic [“non-profit organization”, “role identity of members as producers and consumers”, “principle of solidarity”] features relevant to national individual sports associations. Furthermore, in the specific context of national individual sports associations there are two further characteristics that distinguish them from business organizations, due to their national monopoly position (88) and their restricted access to limited resources within their country. These particularities must also be taken into account in the analysis to enable a well-founded comparison that focuses specifically on national individual sports associations rather than non-profit sports organizations.

Since many of these characteristics are closely interrelated, it is necessary to divide them into different groups for the purposes of the following comparative analysis. This allows for a differentiated consideration of individual aspects and an assessment of their combined effect in the overall context. The following categories, therefore, form the basis for discussion, in which the differences between business organizations and national individual sports associations are discussed in terms of their impact on the transferability of the theory of first-mover advantage:

• Profit- vs. non-profit characteristics
  ◦ Profit maximization vs. orientation towards member's interests
  ◦ Contractual membership vs. voluntary membership
  ◦ Role identity of members as producers vs. role identity of members as producers and consumers
  ◦ Purchasing power of consumers vs. principle of solidarity
  ◦ For-profit organization vs. non-profit organization
• Governance structure
  ◦ Centralized decision-making structures vs. democratic decision-making structures
• Type of employment
  ◦ Paid work vs. voluntary work
• Market structure and competition
  ◦ Position within competitive market vs. monopoly position
• Resource allocation
  ◦ Opportunity of resource availability vs. restricted access to limited resources

In order to assess the differences within the comparative analysis in terms of their impact on the transferability of the theory of first-mover advantage to national individual sports associations, the following evaluation criteria are used:

• No relevant differences: The analysis reveals that there are no significant differences in the examined area. The transfer of the theory remains possible in principle.
• Differences with functional equivalence: Although the analysis reveals existing differences, these lead to comparable benefits or behavior patterns within the respective system. The differences thus lose their relevance for the transfer of the theory, which means that transfer remains possible in principle.
• Differences with a limiting effect: The analysis reveals existing differences that limit the direct transferability of the theory. In this case, subsequent verification is required:
  ◦ Adjustments possible: Modifications allow the theory to still be transferred.
  ◦ Adjustments not possible: There is a structural exclusion criterion for the transferability of the theory to individual sports.

#### 3.1.1 Profit- vs. non-profit characteristics

As for-profit organizations, business organizations primarily pursue the goal of profit maximization (89). Consequently, employees usually being permanently employed and receiving financial compensation for their role and contributions to the production of goods and/or the provision of services. The distribution of goods is based mainly on the individual purchasing power of consumers.

In contrast, national individual sports associations do not have profit maximization as their primary goal. Instead, they serve as non-profit organizations as the representative of the respective sport, representing its interests to society and [inter]national sport organizations, developing rules and regulations, organizing sport events and competitions, and promoting both grassroots and elite sport (4). Accordingly, the association's objectives are based on the interests of its members. The membership is generally voluntary, while the role identity of the members is complex and reflects a mixture of producers, consumers, decision-makers, and financiers (86). At the same time, produced goods and services are distributed among the members on the principle of solidarity and not based on purchasing power (90). However, academic literature outlines a weakening of the principle of solidarity, in theory at least, caused by the increase in money and power (90). These factors also currently play a significant role for national individual sports associations. At least in theory, this could lead to associations increasingly adopting similar characteristics to companies (90). In addition to the multi-layered role identity of their members, the individual national sports associations also represent a monopsony, as they are the only buyers of their product [resp. their members], by recruiting their top athletes to participate and represent them at major international competitions, such as world championships or the Olympics (88).

As this review examines the applicability of the first-mover advantage to the association level in international individual sports, it focuses particularly on the promotion of elite sport by the respective national sports associations. The performance of nations or national sports associations in international individual sports competitions has mostly been evaluated in studies based on their absolute number of athletes in rankings [e.g., (45, 58)] or the ranking position of their top athletes [e.g., (31, 59)]. Accordingly, in an international competition, both the number of top athletes and their sporting success can be seen as the key performance indicator for success, while the top athletes, as the value delivery mechanism, display the product of the respective nations or the national individual sports associations. Unlike in business, the key performance indicator for success like market share is therefore not based on revenue figures but is derived from these sporting parameters in international competition. This differentiated perspective on product and key performance indicators also shows that the profit maximization approach would not be useful for associations. While promoting top-level sport appears to be the key objective for the production of associations when evaluating their sporting performance in international comparison, it also becomes clear that many other association objectives, which are not focused on promoting top-level sport, nevertheless have a positive influence on it. As an example, promoting grassroots or fostering social exchange can also have positive downstream effects on top-level sports, which can be seen in the increase in consumer capital within a country, the expansion of the talent pool or increased interest from sponsors. Considering the role identity of the voluntary members as consumers and the monopsony position of an association as a buyer, it becomes clear that aligning with the interests of the members is essential for the association to achieve its goals. Accordingly, there are clear differences between business organizations and national individual sports associations, which, however, contribute to a functional equivalence in their effectiveness, as they provide comparable benefits regarding the achievement of objectives within their respective systems. Thus, business organizations and national individual sports associations operate in completely different systems, which are, however, each focused on consumers and buyers and designed to achieve the respective objective, which is why these differences do not represent an exclusion criterion for the transfer of the first-mover advantage theory.

#### 3.1.2 Governance structure

In this section, we discuss the different decision-making structures of business organizations and national individual sports associations in relation to their impact on the transferability of the first-mover advantage. In business organizations, authority is typically delegated from the top down through centralized decision-making structures. The formal distribution of power often correlates with the amount of capital invested.

In national individual sports associations authority is generally based on a bottom-up approach whereas the board is elected by all members (86, 88). However, it can be empirically observed that association members only make limited use of the opportunity to participate in democratic decision-making, whether in elections or meetings (85, 91). In addition, there are theoretical approaches in academic literature that build on the iron law of oligarchy (92) and argue that bureaucratization and the increasing demand of expertise could foster oligarchic structures in sports organizations (85). So far, however, these theories have not been confirmed by empirical studies (91).

For the present discourse, it is crucial to evaluate the various structural differences in their entirety. National individual sports associations pursue a wide range of objectives that often can positively influence one another. Consequently, these associations benefit from democratic decision-making structures. These structures enable to fully consider the complex role identity of their members, who also take on the role of consumers, while supporting the different objectives. Accordingly, the decision-making structures of national individual sports associations differ fundamentally from those of business organizations, which, however, has its roots in the different systems [including objective structures and role identity] in which associations and business organizations operate. Yet, this does not represent an issue in terms of transferring the theory. The differences in the decision-making structure between business organizations and national individual sports associations lead to functional equivalence in their effectiveness, as they provide comparable benefits regarding the achievement of objectives within their respective systems. The transfer of the theory is therefore not influenced by these differences.

#### 3.1.3 Type of employment

Another key distinction lies in the different types of employment. In business organizations, employees enter into an employment relationship with their employer, which guarantees them regular salary payments for the work they perform. The basis of this relationship is an employment contract that sets out all rights and obligations.

Opposed to this stands the constitutive feature of voluntary work within the association system (86), according to which sports associations frequently rely on voluntary contribution. This voluntary character does indeed represent a major difference to business organizations.

While voluntary work is widely spread among associations, our analysis focuses on the largest and most important national sports associations in each country, measuring their performance based on sporting success in elite sports. These associations are facing significant challenges arising from globalization, a sharp increase in international competition at elite level, growing expectations of member organizations and external stakeholders, the impact of modern communication and media, the increasing number of partners within the public and private sector, and the need to secure state funding as non-profit organizations (4, 93, 94). In order to ensure their long-term success, many of these sports associations are therefore working to professionalize their structures, with volunteers increasingly being replaced by full-time employees (4, 93). Having professional association structures also creates greater capacity for expanding the member base and promoting elite sports, while also increasing the chances to attract sponsors, which leads to greater autonomy from government funding (95). This professionalization process is not limited to administrative or organizational roles within the associations. Accordingly, a high income and a permanent employment contract are particularly important for coaches in elite sport when considering an international job move (96). This shows that, especially for elite sport, on which this analysis focuses, the structures of the associations are highly professionalized and thus similar to those of business organizations. Due to the similarities outlined, the theory is not excluded from being transferable.

#### 3.1.4 Market structure and competition

In this section, we analyze how the different market structures in which national individual sports associations and business organizations usually operate affect the transferability of the theory. In general, the majority of business organizations operate in open market structures. These are characterized by many suppliers and free competition.

In contrast, national sports associations occupy a monopoly position (88) within their national borders which is a significant difference to business organizations. Consequently, when transferring the theory of the first-mover advantage, it must be questioned whether this analogy would be imperfect for this reason. Accordingly, a first-mover advantage for national individual sports associations might be much stronger as they maintain their monopoly position in their national markets. Furthermore, the global competition is entirely different, since a national individual sports association does not enter the national market of another, whereas the performance comparison in international competitions takes place between individual athletes and not between the associations themselves.

Although national individual sports associations maintain their monopoly position for their sport within their national borders, they are in constant competition for limited resources such as sponsors, talents, spectators, media attention or state funding, with other national sports associations of their country. Accordingly, a sport-specific monopoly may exist for national sports associations, but at the same time they are in a highly competitive environment within the country for relevant resources, which have a significant influence on sporting performance in international competition. Thus, national individual sports associations initially enter an upstream competition for limited resources at a national level, which serve as a basis for the subsequent international sporting competition. Despite their monopoly position, national individual sports associations must therefore also face national competition, although it exists in a different nature. Furthermore, the additional potential difference on global competition whereas individual national sports associations do not enter the national market of another association, is only partially correct. This may be true for a small part of sporting aspects such as the participation in the national sporting league system, which in turn is part of an association and not a relevant aspect when investigating international sporting success, or access to the local talent pool [covered in subsequent section], but it does not apply to the recruitment of employees (96) or even companies for strategic partnerships [e.g., (97)]. In summary, we can state that the permanent monopoly position of national individual sports associations undoubtedly represents a significant difference. However, as explained, these associations also face national competition alongside other associations within their country. This difference therefore does not lead to different patterns of behavior and can be disregarded regarding its relevance for the theory application.

The execution of international sporting competitions by individual athletes and not by national individual sports associations themselves is thereby similar to the competition within the economic system. As already explained, transferring the first-mover advantage to international individual sports competition would mean, that the top athletes represent the product while the number of top athletes or their sporting successes within rankings would represent the key performance indicators of the associations. At the same time, the market in international individual sports consists of the entirety of sporting events, with their outcomes being reflected in the respective world rankings. This is also supported by numerous studies that examined international success in individual sports and based their analyses on world ranking data [e.g., (45, 57–59)]. This approach should guide the theory transfer, as world rankings reflect the athletes' overall yearly performance, which can be used to derive the key performance indicators for individual associations. Within the classical concept of the first-mover advantage, competitive advantages are typically estimated based on market share [e.g., (24, 25)] or operational financial results [e.g., (26)], which are generally derived from an organization's product sales performance within a business year. The representation of an association by its athletes in international competitions is therefore not a limiting factor in transferring the first-mover advantage to individual sports, as it does not reflect a significant difference from deriving key performance indicators within the business world.

#### 3.1.5 Resource allocation

Another clear disparity emerges in how limited resources can be accessed. In general, open market structures allow business organizations to purchase essential but limited resources needed to produce their goods from outside the country [e.g., raw materials].

In contrast, there are essential limited resources for national sports associations in form of the human capital stock (98) and thus the talent pool, which are restricted by the country's own structures. Due to the professionalization in elite sport previously outlined, this limitation primarily affects the talent pool, which is limited for national individual sports associations in terms of the size and age structure of the respective population. In addition, a country's individual economic wealth contributes to the size of the talent pool, as a population can devote more time to sporting activities as its wealth increases (98). These circumstances lead to national individual sports associations facing unequal conditions and resources in an international context, which can affect the sporting competition and therefore also the analysis of the first-mover advantage. Although there are examples of individual athletes changing their national association to represent another country [e.g., (99, 100)], such cases are highly exceptional. While these examples illustrate that elite athletes do not necessarily represent their home country or home association, athletes generally do not have the freedom to choose which national sports association they want to represent within a particular individual sport.

In this case, the comparison reveals differences with a limiting effect. Accordingly, it is necessary to further explore if a modification can enable the transfer of the theory. In order to take these significant differences into account when transferring the theory to the association level in individual sports, two key aspects must be considered. Firstly, the restricted access to essential but individually different limited resources must be considered when transferring the first-mover advantage. Since countries differ fundamentally in terms of population size, age structure, and wealth, the size of the respective talent pool also varies. Therefore, when conducting an empirical study on the first-mover advantage in individual sports, macroeconomic control variables of countries, such as population size or GDP [per capita], should be included to account for these unequal conditions and to avoid possible distortions. Secondly, as athletes do not necessarily represent their home country or home association, the work of the national associations in developing the athletes needs to be considered in the analysis as well. Accordingly, athletes need to be assigned to the individual national sports associations prior to the analysis, based on the contribution of the associations to the development of the respective athlete, not based on the flag they currently represent. The implications outlined are a necessary requirement to account for the restrictive differences between business organizations and national individual sports associations in order to enable a fundamental applicability of the theory of the first-mover advantage to the association level in individual sports.

Table 3 provides a summary of the structural differences between business organizations and national individual sports associations and highlights their impact on the transferability of the first-mover advantage to individual sports.

**Table 3:** Summary of the structural differences and their impact on the transferability of the first-mover advantage to individual sports.

Structural characteristics	Business organization	National individual sports association	Impact on transferability of the first-mover advantage
Profit- vs. non-profit characteristics	– Goal: profit maximisation – Membership: contractual and compensated by default – Role identity of members: producer by default – Distribution of goods: based on individual purchasing power of consumers	– Goal: based on the interests of its members – Membership: on voluntary basis – Role identity of members: producer, consumer, decision-maker and financier – Distribution of goods: among members on principle of solidarity	Difference does not affect transferability
Governance structure	Hierarchical decision-making structure—top-down approach	Democratic decision-making structure—bottom-up approach	Difference does not affect transferability
Type of employment	Overwhelmingly full-time employees with recurring salary payments	Voluntary work is typical/widespread among sports associations; however national sports associations are increasingly professionalized	Difference does not affect transferability
Market structure and competition	Open competition in national and international markets, usually with a large number of competitors	National monopoly. Competition takes place through sporting events by elite athletes of the associations	Difference does not affect transferability
Resource allocation	Generally, no differently restricted access to resources between business organizations	Differently restricted access to essential limited resources	Difference requires adjustments

Source: Own table.

### 3.2 Theoretical modeling: elements of the first-mover advantage and their theoretical transferability from corporate to association level in individual sports

The theory of first-mover advantage was first conceptualized and published by Lieberman and Montgomery in 1988. Lieberman and Montgomery (21) stated that the first-mover advantage for pioneer companies results from technological leadership, generating product-related switching costs that consumers incur during a purchase decision when switching to a competing product, and the initial access and securing of limited resources [e.g., human capital]. These asymmetric competition conditions for early market entry allow the pioneer to create market entry barriers, which can sustainably strengthen its market position against subsequent competitors (101, 102). However, Lieberman and Montgomery (21) also identified potential disadvantages of early market entry, as following companies can learn from the pioneer's mistakes, benefit from its investments, and are not exposed to the risk of the pioneer's technological and market uncertainties. This makes it much easier to react to rapid changes in technology or consumer behavior (103) and facilitates the market entry in general. In a systematic literature review of studies on first-mover advantage, Tsuchihashi (104) was able to show that most studies indicate a positive effect of the first-mover advantage.

According to the current state of research, the theory developed by Lieberman and Montgomery, including its components, forms the basis for theoretical modeling.

Within the theoretical modeling, we transfer the three elements of the first-mover advantage to national individual sports associations to analyze if these components would also be applicable to individual sports at an international level. In this context, the founding year of the national individual sports associations serves as the indicator for the first-mover advantage. The operationalization is based on the founding year, as the founding marks the beginning of the organized development of sport-specific structures within a country and simultaneously creates the possibility that athletes supported by the association and representing the association can be ranked in the world rankings for the first time, which corresponds the concept of market entry of companies in an economic context. This approach is in line with previous research on the first-mover advantage in team sports (20, 22).

#### 3.2.1 Technological leadership

As the governing body, national sports associations are responsible, for example, of talent development, training and education of coaches, and promoting exchange between their members (88). To fulfil these tasks, professional structures need to be created and established to ensure the long-term development of the sport within the country.

Therefore, a pioneering national individual sports association would enable its members to be the first to enter into an exchange within these structures, thus initiating a sport-specific learning process. This could lead to a knowledge advantage in many essential sport-specific areas and ultimately result in a technological leadership position.

#### 3.2.2 Generation of product-related switching costs

Business organizations can create market entry barriers by investing in strategies that significantly increase the resource requirements of competing firms (105). At the same time, a central strategic task of national sports associations lies in the promotion of the respective sport (4, 88). This is done, for example, by initiating strategic programs for the attraction and retention of athletes, which in turn are implemented by local clubs and coaches (46).

The pioneer move would give a national individual sports association the opportunity to launch initial nationwide promotional programs to attract athletes at the earliest stage and retain them long-term. If the pioneering association implements its programs on a large scale, the athletes could develop an intense connection to the sport. As a result, subsequent national sports associations in the country would have to invest significantly more resources to motivate athletes to switch sports. This reduces the likelihood for the pioneering association that an athlete will quit or switch sports, which would reveal the emergence of product-related switching costs.

#### 3.2.3 Initial access and securing of limited resources

National individual sports associations are faced with a number of limited resources, such as athletes [due to the limited national talent pool], coaches, medical staff and infrastructure, sports infrastructure, or sponsors.

In this context, a pioneer move would enable a national individual sports association to be the first to access these limited resources, allowing the pioneer association to secure and utilize these resources to its advantage early on. Therefore, from a theoretical perspective, this element of the first-mover advantage can be applied to the level of national individual sports associations.

#### 3.2.4 Summary of theoretical modeling and further indications

Having examined the individual first-mover advantage components, the following summarizes their collective outcome and reinforces its relevance through concrete examples. The theoretical modeling of the transferability of the individual first-mover advantage components to the level of national individual sports associations, based on the founding year of the respective association, shows that, at least in theory, the individual components could also exist for national individual sports associations. This indicates that a first-mover advantage represents a theoretically plausible phenomenon that could occur in individual sports. The results of theoretical modeling can be supplemented by economic findings. For example, there is some research on first-mover advantage that shows positive effects for companies with structural similarities to national individual sports associations. In terms of their production objective and product type, national individual sports associations are particularly similar to business organizations in the service sector, such as private flight or driving schools, which train pilots or drivers as service providers. At the same time there are numerous studies in the academic literature that provide evidence of a first-mover advantage in the service sector (24–26). On top of that, there is scientific evidence that, for businesses with limited resources in international markets, early market entry is crucial for success (27). In addition to these parallels, there are aspects within individual sports itself that can be interpreted as indicators pointing to the existence of the first-mover advantage. Accordingly, there are some individual sports in which especially older national individual sports associations are very successful in international comparison. A prominent example is tennis, where France [number of top 100 athletes: 13; association founded: 1888], the United States [9; 1881] and Australia [9; 1904], all of which are among the oldest national tennis associations, currently count the most ATP athletes in the top 100 of the ATP singles rankings (28). This relation is also observable in golf. Within the Official World Golf Ranking, the United States [association founded: 1894] and Canada [1895], two nations with comparably old national golf associations, represent more than half of the athletes in the top 100, with the United States alone accounting for 47 athletes (29).

### 3.3 Case logic: analyzing potential effects through the theoretical transfer of the first-mover advantage to individual sports

The case logic provides a theoretical examination of the potential effects that an existing first-mover advantage could have on national individual sports associations. The aim is to analyze if and how a first-mover advantage effect would be observable for individual sports associations. The analysis is based on the individual components that define the first-mover advantage (21).

#### 3.3.1 Technological leadership

The theoretical transfer of the first-mover advantage of technology leadership reveals several areas that can be positively affected within the pioneering association. Possible effects include advantages in the equipment of training facilities, the development of training concepts, the qualification and training of coaches, the association's sports policy strategy, and the advancement of knowledge and research in the field of sports science and sports medicine. Taking on the pioneering role provides the opportunity to become a leader in each of these areas. This provides a conceptual basis for utilizing specialized expertise to initiate process innovations at an early stage, which in turn strengthens the technological leadership position. While companies experience learning curve effects as production increases, applying the first-mover approach to individual sports allows the company's production to be compared with the practice of the sport and the operational working methods within a national individual sports association. Due to the time advantage over other nations, learning curve effects can be used within the respective areas to expand and strengthen the leading position. In this case, the learning curve effects result through the increased practice of organized sport. Learning curve effects allow national individual sports associations to reduce the average cost of the sport produced as volume increases, leading to lower marginal costs per unit for specialized nations as production volume increases (106). This initiates a continuous improvement process, which is necessarily time-dependent (22). The resulting learning curve effects can appear in the areas mentioned, for example, through an optimized approach of a country's sports policy, lower costs for training, or a greater number of sports facilities, while even the pure availability of sports infrastructure already has a significant influence on sporting activity (107). Likewise, continuous development of sport infrastructure as well as of research in sports science and sports medicine can contribute to providing players with the highest scientific level of training in specialized training centers, ensuring excellence in education and coaching of coaches, and offering superior medical care for athletes. The revealed potential effects of a leadership position in areas such as training facilities, training concepts, coach qualifications, and the sports policy of an association show a clear overlap with the reality of national individual sports associations. In golf, both top-level coaching and professional association structures that provide well-established training programs for clubs play a central role in sporting success (44, 50). In tennis, too, scientific studies show that professional association structures, educational concepts for coaches, top-level coaching, and professional association infrastructure can have a positive influence on international success (42, 46, 47). According to Brouwers et al. (42), aspects like well-trained and experienced elite tennis coaches, a long-term plan for elite tennis development, coordinated planning for the development of talent in tennis, a national coordination plan for tennis facilities, and a network of high quality national and regional elite tennis centers can each have a positive influence on international success in tennis. These are all factors that could be positively influenced by a pioneering move and the resulting leadership position.

#### 3.3.2 Generation of product-related switching costs

Applied to international individual sports, the first-mover advantage of generating product-related switching costs becomes visible through the early creation of consumer capital on the demand side and the establishment of a sport-specific culture within the pioneer's country. Given the lack of sports opportunities, the pioneer can meet a corresponding demand when entering the market. Through the consumption of the respective individual sport, sport-specific know-how develops within the consumer level, which increases the identification with the sport and reduces the risk of consumers finding a comparable benefit in another sport (22). The pioneer move therefore enables an early promotion and development of consumer capital in the country. At the same time, the pioneering association can reduce the risk of athletes switching sports or turning away from the respective individual sport as a result of a strong development of consumer capital. Sporting successes, due to learning curve effects, and the increasing accumulation of consumer capital also strengthen the social significance of the sport, which in turn fosters the formation of a sport-specific culture in the pioneer country. Consequently, consumer capital is also generated through an increased presence in the mass media, while professional athletes gain social status as popular role models (22, 42). In addition to sporting success and strong consumer capital, a country's sport-specific culture can also increase the “product-related switching costs”, as more athletes take up the respective sport and fewer athletes switch sports, leading to an increase in the number of athletes and the size of the talent pool. Accordingly, the pioneer association not only benefits stakeholders in their respective sport but also achieves positive effects across all sports within their own country. The potential effects outlined above are highly relevant for national individual sports associations. As explained in section 3.1, these associations face the challenge of dealing with a limited talent pool, which is restricted by the structural conditions of the respective country and for which they have to compete with other national sports associations within their country. Accordingly, it is standard practice in individual sports for national associations to prioritize the generation of consumer capital. This is achieved in particular by initiating grassroots and youth programs, which are designed to attract talents at an early stage and retain them long-term [e.g., (44, 46)]. In Swedish golf, well-designed and funded youth programs have been identified as a key factor in international success (44). Research on success factors in international tennis reveals that coordinated planning for the development of talent in tennis and a high general tennis participation each can have a positive impact on an association's international tennis success (42). Wijk (55) also identified a positive correlation between the spread and promotion of tennis in the mass media in Sweden and the nation's international tennis success in the 1980s. The generation of consumer capital, for example through early targeted programs, promotes the formation of a sports culture in the country and increases product-related switching costs, which appears essential for national individual sports associations due to their limited talent pool.

#### 3.3.3 Initial access and securing of limited resources

The transfer of the first-mover advantage of initial access and securing of limited resources can be noticed by the opportunity of securing important human resources. In particular, the pioneer move provides the opportunity to be the first to access limited essential human resources. Special relevance can be attributed to limited sport-specific resources such as athletes, sports managers, medical staff, fans, and specialists responsible for the development [coaches] and identification [scouts] of talents. The combination of the advantages outlined so far, such as securing limited human resources, who can continuously develop the improvement process through learning curve effects and the increased social significance through the early establishment of a respective sport-specific culture, opens up the potential to secure important economic resources over time through the growing interest of sponsors. Although the transfer of the first-mover advantage to individual sports is not immediately followed by securing economic resources through sponsorship income as a result of the initial market entry, a time-delayed connection and a positive interaction between securing limited resources, technological leadership, and the generation of product-related switching costs can generally be assumed in this case. The potential effects outlined also align with the reality of individual sports associations. In addition to the already highlighted need to attract and retain limited resources at an early stage, such as talents and qualified coaches, evidence can also be found in individual sports for the relevance of effective systems for the detection of young talent and the associated specialized personnel in scouting (42). As the development of professional athletes can be extremely cost-intensive, sponsorship income plays an important role for both national individual associations and athletes (42, 52, 56). According to the study by Brouwers et al. (42), the various funding components “sufficient financial support for tennis associations”, “sufficient financial support for grassroots tennis”, and “sufficient financial support for elite tennis” each have a decisive influence on international tennis success. Consequently, studies show that sponsors and the financial resources of associations play an important role in international sporting success in individual sports (42, 47, 55, 56), highlighting the parallel to the potential first-mover effects outlined.

#### 3.3.4 Summary of case logic

The theoretical transfer of the first-mover advantage to the level of national associations in individual sports indicates the extent to which the potential pioneer advantage could influence success in individual sports. At the same time, the study findings in individual sports demonstrate that potential effects of first-mover advantage influence areas that have been identified as contributing to sporting success. The practical examples therefore underscore the fundamental need to examine the transferability of the theory of first-mover advantage. Regarding the classification of success factors, it appears that a potential first-mover advantage would affect the overlapping level between the macro and meso levels as well as the meso level. Looking more closely, it becomes clear that the potential first-mover advantage in individual sports could have an impact on all previously identified success factors at these levels. Therefore, already identified success factors such as top-level coaching, the sports policy of an association, or the extent of the respective sport-specific culture within the country, could be long-term effects of a first-mover advantage. However, the first-mover advantage itself would be categorized as a success factor at meso level, based on the definitions provided by De Bosscher et al. (38).

## 4 Discussion

The previous theory adaption examined the possibility of transferring the theory of fist-mover advantage to the international association level in individual sports from three different perspectives. To answer the research question, a final discussion of the findings obtained through our study is required.

The critical review of the differences between business organizations and national individual sports associations within the comparative analysis leads to the conclusion that the identified structural differences do not preclude a transfer of the first-mover advantage. However, when transferring the first-mover advantage, macroeconomic control variables would have to be included in the study to consider each association's individually restricted ability to access essential limited resources. Furthermore, the assignment of athletes to the respective national individual sports associations would have to be based on their impact on the development of the athletes to take into account the possibility of athletes changing associations.

Within the theoretical modeling we discovered that the individual components of the first-mover advantage can generally be transferred to the national association level in individual sports. The theoretical transfer of the first-mover effect, which was part of the case logic, provides indications, according to which a plausible first-mover effect can generally exist in individual sports. Accordingly, a potential effect could be generated primarily by positive impact potentials within the areas of consumer capital [e.g., formation of a sport-specific culture, larger talent pool, increased attractiveness for sponsors], human capital [e.g., securing limited resources, knowledge and performance advantage through learning curve effects], and superior infrastructure. As practical examples show, the potential effects outlined are highly relevant to the reality of national individual sports associations.

Bearing in mind the requirements outlined, we conclude that the theory of first-mover advantage is generally transferable to the national association level in individual sports.

**Figure 1:**
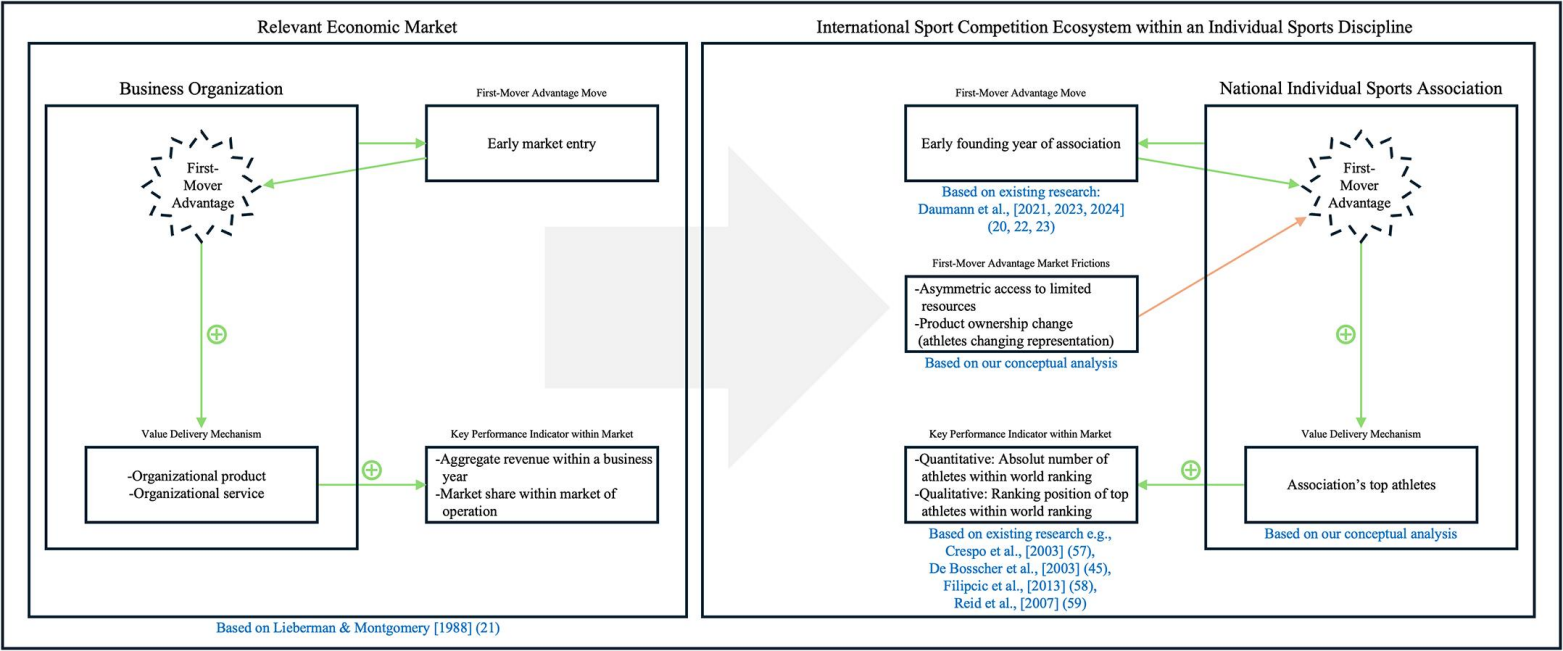
Conceptual model illustrating the transfer of the first-mover advantage theory to national individual sports associations. Own figure.

Figure 1 presents our model, which illustrates the transfer of the first-mover advantage theory to association level in individual sports.

A potential boundary could only exist for individual sports in which athletes can change their national affiliation and association with little effort and high frequency. In such sports, the theory of first-mover advantage would not be transferable, as athletes take on the role of the product within the theory transfer, which, in an economic context, does not change arbitrarily between business organizations. If such individual sports disciplines exist, needs to be clarified in advance of empirical research based on the career paths of athletes.

Our analysis of the transferability of the first-mover advantage theory to individual sports also provides indications that a first-mover advantage could exist in individual sports. Accordingly, empirical studies provide evidence of a first-mover advantage for business organizations that share structural characteristics with national individual sports associations (24–27). The simulated potential effects of early market entry by national individual sports associations also show high practical relevance, whereas in individual sports such as tennis and golf, athletes from countries with established associations dominate the respective world rankings (28, 29). To deepen the current findings, future studies should therefore explore if the first-mover advantage actually exists in individual sports. Accordingly, we were able to identify several potential research areas for future studies on the first-mover advantage among national individual sports associations. Equivalent to studies in team sports (20, 22), future studies should analyze the first-mover advantage within international competition in an individual sporting discipline [e.g., tennis or golf]. To investigate a fundamental effect, a broad-based empirical analysis over an extended observation period is recommended. In this case, sporting performance could be operationalized using world ranking data, while applying the same observation years from team sports analyses would enable meaningful comparisons of the results. The analysis could be carried out using a random intercept model, for example. A possible hypothesis for investigating a potential first-mover effect in individual sports could be as follows:

H: The earlier a national individual sports association was founded, the more successful it is [e.g., measured by the number of athletes in world rankings or cumulated position of the best *n* athletes within world rankings].

**Figure 2:**
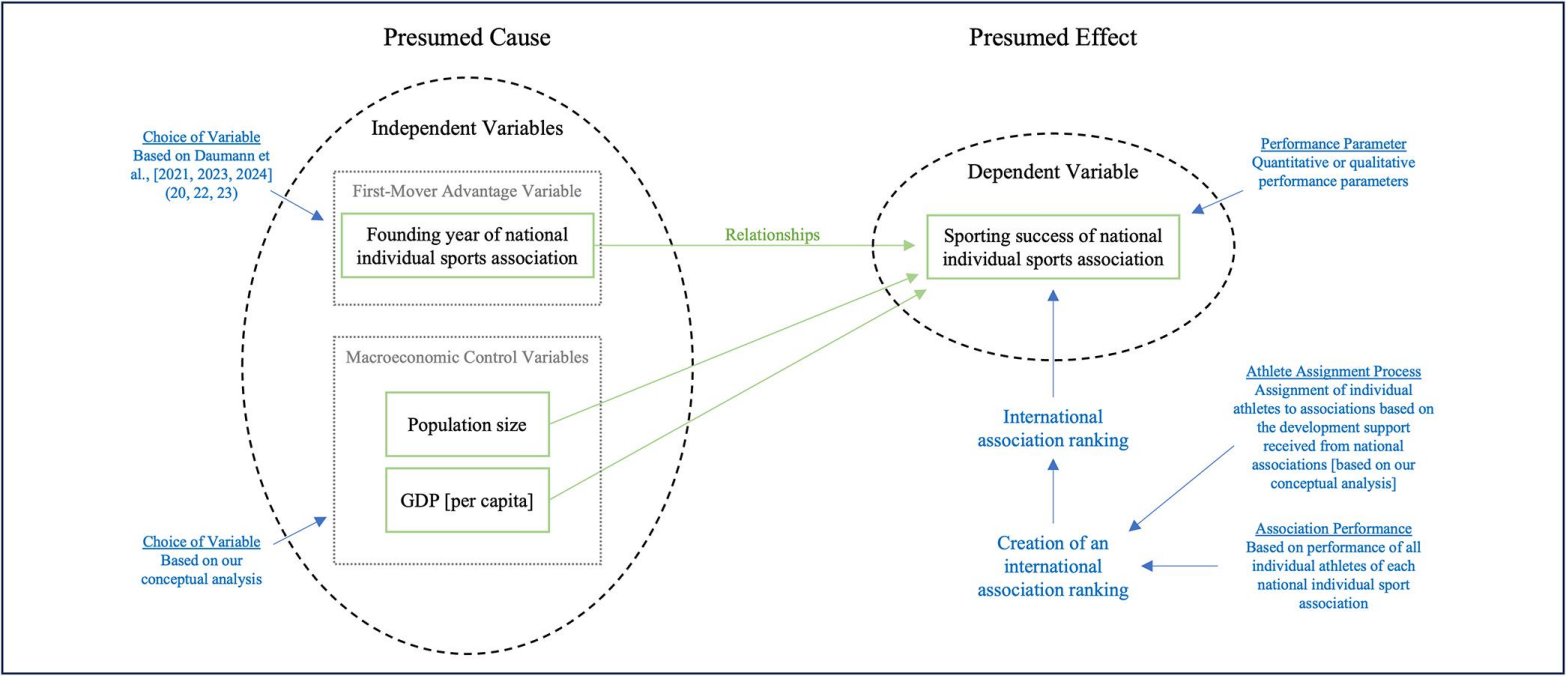
Conceptual framework for the transfer of the first-mover advantage to association level in individual sports. Own figure.

To bridge the gap between theory and practice, Figure 2 provides a comprehensive overview of the conceptual framework to transfer the first-mover advantage to the level of national individual sports associations.

If a first-mover effect can be empirically identified, further studies could be conducted, for example in the form of a case study or again in a multilevel analysis, to specifically explore which of the three components of the first-mover advantage are primarily responsible for these positive effects. These findings could then be used to develop valuable success-focused implications for action. Considering macroeconomic control variables, potential hypotheses for the respective first-mover advantage components could be as follows:

H1: National individual sports associations that were first to establish national training centers are more successful long term [e.g., measured by the number of athletes in world rankings].
H2: National individual sports associations that are first to implement national talent development programs are more successful in building a larger talent pool per capita.
H3: National individual sports associations with a higher number of licensed coaches per capita are more successful long term [e.g., measured by the number of athletes in world rankings].

Despite existing empirical evidence on first-mover advantage in team sports (20, 22, 23), our conceptual work represents the first theoretical examination of its transferability to sport. A systematic theoretical foundation for transferability and a corresponding theoretical framework have not yet been developed. Since the first-mover advantage is closely linked to strategic behavior and management (98), it appears that the outlined general differences in sport production between individual and team sports are less relevant. The close links in management and strategic direction are also evident in numerous studies that deal with sports governance, sports policy, and sports management in an international context and do not differentiate between individual and team sports [e.g., (4, 18, 38–41, 108)]. Due to their comparable structural conditions, the results of the comparative analysis and the theoretical modeling can also be applied to national team sports associations. The theory of first-mover advantage is therefore applicable to both national associations in individual and team sports. Our work thus anchors existing empirical research findings in team sports, while at the same time it differentiates itself. Accordingly, differences in production, which are reflected, for example, in the generally lower costs of education and training in the empirically studied team sports of soccer and rugby compared to golf and tennis, limit the applicability of all findings. As a result, the potential effects highlighted in the case logic are derived specifically from the circumstances of the individual sport, which differentiates the work.

This paper takes a conceptual approach to examine the theoretical transferability of the first-mover advantage to individual sports. The key limitation of this study lies in the fact that the findings do not reveal any conclusions about an actual first-mover advantage in individual sports. Therefore, no statement can be made as to if the identified potential first-mover effects actually exist and, if so, for which individual sports. To deepen the current findings, additional research is therefore required.

## 5 Conclusions

The primary objective of this paper was to explore in a conceptual analysis, if the theory of first-mover advantage can be transferred to individual sports. Since the first-mover advantage has already been applied to team sports in several studies that provided empirical evidence of a strong effect (20, 22, 23), this paper represents another important research contribution by evaluating the transferability to individual sports for the first time. The findings, according to which the theory of the first-mover advantage can generally also be applied to individual sports, under the conditions outlined, serve as an important basis for future studies within this research field. While this paper discusses many structural differences between sports associations and business organizations, the transferability of the theory of first-mover advantage to individual sports highlights the overarching management parallel between decision-making in sports associations and business organizations. Considering the intensifying international competition at elite level and the resulting need to professionalize organizational structures (4), this parallel might be particularly relevant for national sports associations to identify new implications for action in the future.
